# The Contribution of Attention to the Mere Exposure Effect for Parts of Advertising Images

**DOI:** 10.3389/fpsyg.2018.01635

**Published:** 2018-09-05

**Authors:** Yoshihiko Yagi, Kazuya Inoue

**Affiliations:** ^1^Faculty of Psychology, Rissho University, Shinagawa-ku, Japan; ^2^Faculty of Engineering, Information, and Systems, University of Tsukuba, Tsukuba, Japan

**Keywords:** mere exposure effect, attention, advertising, preference, face

## Abstract

Repeatedly presented stimuli are affectively evaluated more positively than novel stimuli. This phenomenon, known as the mere exposure effect, is used in advertising. However, it is still unclear in which part of advertising images the mere exposure effect occurs. Given the recent suggestion that attention plays an important role in the mere exposure effect, it is possible that the mere exposure effect does not occur for commercial products when advertising images consist of a commercial product along with an attractive human model. To investigate this possibility, we manipulated the relationship between advertising images repeatedly presented in an exposure phase and images presented in a later rating phase. In the exposure phase, participants were repeatedly presented with advertising images consisting of a cosmetic product along with an attractive female model and were instructed to attend to a specified part of the image (Experiment 4) or were given no such an instruction (Experiments 1, 2, and 3). In the rating phase, participants were asked to evaluate their preference for complete advertising images (Experiment 1), the images of female models (Experiment 2), or images of products (Experiments 3 and 4) that were previously presented or not presented. The mere exposure effect was found for whole advertising images and images of female models. On the other hand, the mere exposure effect for the images of products was seen only when participants were explicitly encouraged to direct their attention to the product parts of the advertising image. That is, the results of this study suggest that the mere exposure effect does not always occur for every part of the repeated advertising images, and that attention would modulate the mere exposure effect for advertising images.

## Introduction

Almost all commercial companies use advertising to increase sales of their products. According to one advertising model, the dual mediation hypothesis (MacKenzie et al., 1986), attitudes toward advertisements influence a customer’s intention to purchase through affecting both attitude toward brands and brand cognition. Several mechanisms are involved in changing attitudes through advertising, such as evaluative conditioning (Stuart et al., 1987; Pleyers et al., 2007) and self-referencing (Lee et al., 2002). Another important mechanism in attitude change is the mere exposure effect (Zajonc, 1968), in which repeatedly presented stimuli are evaluated more positively than novel stimuli. In fact, previous studies have demonstrated that repeated presentation of banner and television advertisements increase positive attitudes toward the advertisements (Fang et al., 2007) and advertised brands (Yoo, 2008).

To facilitate the application of the mere exposure effect on advertising, it is important to clarify the characteristics of the mere exposure effect in terms of human factors. For this purpose, an important factor for consideration is selective attention. Several studies have demonstrated the importance of selective attention in affective modulation of exposed stimuli (Raymond et al., 2003; Yagi et al., 2009; Huang and Hsieh, 2013). For example, Yagi et al. (2009) reported that the supraliminal mere exposure effect occurred only for an object selected by attention. Here, participants were repeatedly presented with a composite figure consisting of a red polygon and a green polygon. The participants were required to attend to either or both of the colored components. Results showed that the mere exposure effect was obtained only for the polygons morphologically identical to the previously attended polygons. The effect of selective attention on affective evaluation has also been reported in studies of the distractor devaluation effect (Raymond et al., 2003), in which previously unattended objects are devalued relative to previously attended objects or novel objects. The results of these studies contradict the naïve intuition that the mere exposure effect always occurs for every part of a previously exposed complex stimulus.

The fact that the mere exposure effect cannot always occur for every part of complex stimuli is especially important in terms of change of attitude through advertising imagery. Advertising images often contain commercial products as well as celebrity or attractive models (Sliburyte, 2009). Thus, if the attractive model, rather than the commercial product, captures a viewer’s attention it is possible that the mere exposure effect does not occur for commercial products but for the attractive models, which would be undesirable for the sponsor of the advertisement. This phenomenon is likely to occur given the previous studies of attention. For example, human faces obligatorily captured attention even though the faces were unrelated to the task required for participants (Sato and Kawahara, 2015). In addition, attractive faces were more difficult to ignore than unattractive faces even though these faces were task-unrelated (Sui and Liu, 2009).

The aim of the present study is to clarify what part of an advertising image triggers the mere exposure effect. To achieve this aim, we mainly manipulated the relationship between advertising images repeatedly presented in an exposure phase and images presented in a later rating phase. In the exposure phase of all experiments, participants were repeatedly presented with advertising images consisting of multiple objects, specifically, a commercial product and an attractive, female model. In the rating phase of Experiment 1, participants were asked to evaluate their preference for the exposed advertising images and for unexposed advertising images. The aim was to ensure whether the mere exposure effect occurred for the exposed advertising images themselves. In contrast, in the rating phase of subsequent experiments, a female model (Experiment 2) or a product (Experiment 3) previously included in the exposed advertising images was presented to investigate whether the mere exposure effect can occur for only a part of the advertising images. We also replicated Experiment 3 with additional instructions that encouraged participants to direct their attention in the exposure phase to the part of the advertisement containing the product (Experiment 4). This was done to explore the effect of selective attention on, if any, differences in the mere exposure effect between Experiments 2 and 3.

Note that, contrary to Yagi et al. (2009), we did not explicitly manipulate selective attention in the exposure phase of Experiments 1, 2, and 3 because in everyday life people often view advertising images without specific intentions. It is expected that these experiments will extend the implications from Yagi et al. (2009) to the domain of consumer psychology, which should provide useful knowledge to development of the effective design of advertising images.

## Experiment 1

The aim of Experiment 1 was to investigate whether the mere exposure effect occurs for the images themselves in advertisements. Understanding this process is important because a recent study suggested that the mere exposure effect occurs only for stimuli whose initial pleasantness is moderate (Delplanque et al., 2015). Therefore, advertising images with an attractive female model may be so positive that the mere exposure effect would not occur. Moreover, this experiment would provide fundamental data to ensure the mere exposure effect occurs for the advertising images used in the present study.

### Method

#### Participants

Twenty-five (14 males and 11 females) adults participated in this experiment for 300 yen (mean age = 19.08 years, *SD* = 1.06). All participants had normal or corrected-to-normal vision.

#### Apparatus and Stimuli

Stimulus presentation and response collection were controlled by a laptop computer (ZenBook UX31E, ASUSTeK Computer Inc.) running Windows 7. All stimuli were presented on the display of the laptop computer at a resolution of 1024 × 768 pixels, with a viewing distance of 57 cm.

**FIGURE 1:**
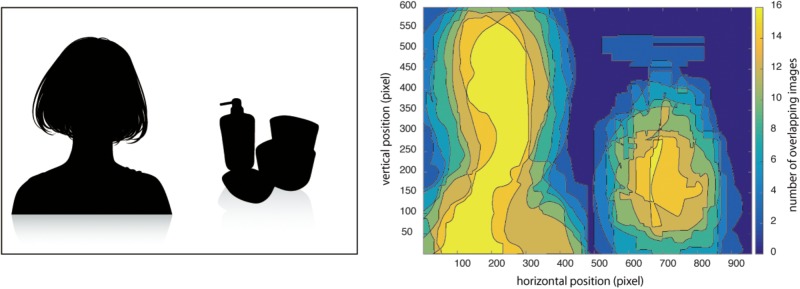
A schematic illustration (left) and the contour graph (right) of the advertising images used in the experiments. To create the contour graph, advertising images in which a female model was located on the right side of the image were reflected along the vertical axis. Then all pixels of each image were binarized (0 as the background containing neither a female model nor a product and 1 as the area containing either the model or the product) and all images consisting of binary values were combined into the image of the contour graph. The higher (lower) contour value and more yellowish (bluish) colors indicates areas that had the greater (lesser) overlap of female models or products across the images.

Sixteen published advertising images were obtained from the Internet (**Figure 1**). Width and height were 960 and 600 pixels, respectively. Each advertising image consisted of a cosmetic product along with a female model. It is known that over exposure diminishes the mere exposure effect (Bornstein et al., 1990). Therefore, models that were unfamiliar to Japanese participants were taken from Chinese and Korean advertisements. The left (right) half area of the advertising images contained the female model whereas the remaining half area contained the cosmetic product. Based on results of the pilot study, the advertising images were divided into two sets, each with approximately the same values of preference.

#### Procedure

All procedures in this and following experiments were approved by the local ethics committee of the Faculty of Psychology, Rissho University. In accordance with the ethics code of the American Psychological Association, at the beginning of the experiments, participants were informed of the expected duration of the experiment and their right to decline or withdraw from the experiment.

Experiment 1 consisted of an exposure phase and a rating phase. In the exposure phase, each trial began with the presentation of a fixation point for 500 ms, immediately followed by the presentation of an advertising image for 500 ms. After the removal of the advertising image, a blank display was presented for 1500 ms, which was followed by the fixation point for the next trial. Participants were asked to focus on the advertising image during a trial. The exposure phase consisted of 64 experimental trials as follows. Each participant was assigned to one of the two sets of eight advertising images; assignment was counterbalanced across participants. Each advertising image was presented eight times in random order, with the restriction that the same image never appeared on three consecutive trials.

In the rating phase, a fixation point was presented for 500 ms, immediately followed by the presentation of an advertising image along with a 5-point rating scale ranging from 1 = not preferable to 5 = preferable. The participants were asked to rate preference for the advertising image on the scale by pressing a corresponding numerical key. The advertising image remained on the display until the participant’s key press. The rating phase consisted of 16 trials. In half of the trials, advertising images presented in the exposure phase appeared (i.e., the exposed condition) whereas in the remaining trials novel advertising images not presented in the exposure phase appeared (i.e., the novel condition). The presentation order of the advertising images was completely randomized across participants. After finishing the rating phase, participants were asked to report whether they noticed the purpose of the experiment throughout the experiment.

### Results and Discussion

**FIGURE 2:**
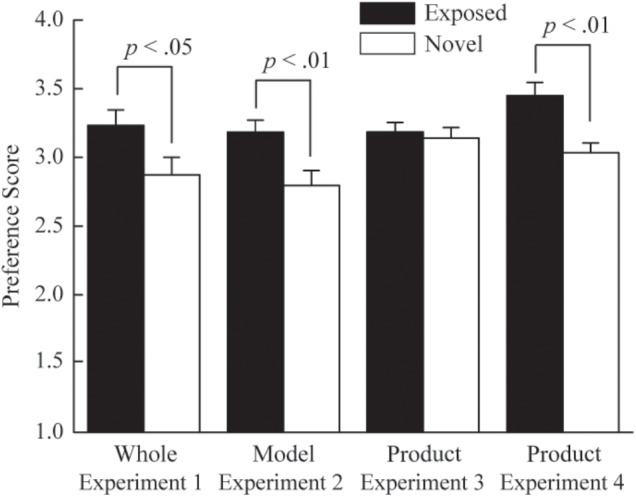
Mean preference scores in Experiments 1–4 (Error bars are MSE). The black and white bars indicate the results in the exposed and novel conditions, respectively. The labels of the x-axis denote the experiment number and objects that were evaluated.

We excluded participants that were aware of the purpose of the experiment from all the data analyses to avoid demand characteristics, such as participants’ responses to fulfill the expectations of the experimenter. In Experiment 1, one participant became aware of the purpose of the experiment and was excluded from the following analysis. **Figure 2** shows the means of preference scores for the exposed and novel conditions. We used a two-tailed paired *t* test to compare the exposed condition with the novel condition, which showed that the mean preference score was significantly higher in the exposed condition, compared to the novel condition, *t*(23) = 2.36, *p* = 0.03, *d*_z_ = 0.48, indicating that the mere exposure effect occurred in Experiment 1. That is, results of Experiment 1 suggest that repeated presentation of complex advertising images consisting of a product along with a female model, induces the mere exposure effect for the exposed advertising images themselves.

## Experiment 2

The aim of Experiment 2 was to investigate the mere exposure effect for the female model part of advertising images. Participants were repeatedly presented with whole advertising images in the exposure phase and were asked to rate preference for the images of female models in the rating phase.

### Method

#### Participants

Thirty-nine adults (23 males and 16 females) participated in this experiment. None of these participants had participated in Experiments 1. All participants had normal or corrected-to-normal vision.

#### Apparatus and Stimuli

The advertising images in the exposure phase were the same as those used in Experiment 1. However, the stimuli used in the rating phase of this experiment were the images of female models clipped from the advertising images. The latter stimuli were simply created by dividing each advertising image into to two parts (i.e., female model and product parts) at the center. Thus, the width and height of the images of female models were 480 and 600 pixels, respectively. Based on results of the pilot study, the images of 16 female models were divided into two sets, each with approximately the same preference values. The corresponding advertising images for the exposure phase were also divided into two sets, based on the division of the images of female models.

#### Procedure

The procedure was identical to that of Experiment 1, with the following exceptions. In the rating phase, the participants were presented with images of female models but not with the whole advertising images. Participants were asked to rate their preferences for the female models using a 5-point scale (1 = not preferable and 5 = preferable). The rating phase consisted of 16 trials. Half of the female models (8 trials) were included in the advertising images previously presented in the exposure phase (the exposed condition) whereas the images of the other models (8 trials) were not included in the exposed advertising images (the novel condition). The assignment of each set of female images to each exposure condition was counterbalanced across the participants. The presentation order of the female images was randomized for each participant.

### Results and Discussion

One participant who rated all images of the female models as 1 was excluded from the following analysis because of the possibility that the participant did not follow instructions correctly. We conducted a *post hoc* power analysis for the paired *t* test based on the effect size in Experiment 1, the sample size of this experiment, and a significance level of α = 0.05 to determine if this experiment had sufficient statistical power (Faul et al., 2007). The obtained power was 0.82, which exceeded the common criterion of 0.80 suggested by Cohen (1992). Therefore, this experiment was considered to have sufficient power to reliably identify the effect of mere exposure.

**Figure 2** shows the means of preference scores for the exposed and novel conditions. Again, the paired *t* test indicated that the mean preference score in the exposed condition was higher than in the novel condition, *t*(37) = 3.49, *p* < 0.01, *d*_z_ = 0.57. This result suggests that when complex advertising images consisting of a product along with a female model are repeatedly presented, the mere exposure effect occurs for a component (i.e., female models) of the advertising images.

## Experiment 3

The aim of Experiment 3 was to investigate the mere exposure effect for the product parts of repeatedly presented advertising images. Given that the significant mere exposure effect could be seen for the section of the ads with a model in Experiment 2, there is no reason that visual change between the exposure phase and the rating phase *per se* would diminish the mere exposure effect in this experiment.

### Method

#### Participants

Thirty-eight adults (22 males and 16 females) participated in this experiment (mean age = 20.08 years, *SD* = 0.88). None of these participants had participated in Experiments 1 or 2. All participants had normal or corrected-to-normal vision.

#### Apparatus and Stimuli

The advertising images in the exposure phase were the same as those used in Experiments 1 and 2 whereas the stimuli used in the rating phase differed from those in the two prior experiments. In Experiment 3, the images of cosmetic products clipped from the advertising images were used. These stimuli were created by the same manner as those of Experiment 2. Thus, the width and height of the images of cosmetic products were 480 and 600 pixels, respectively. The images of 16 cosmetic products were divided into two sets, each with the approximately same values of preference. The corresponding advertising images for the exposure phase were also divided into two sets, based on the division of the images of cosmetic products.

#### Procedure

The procedure was identical to that of Experiments 1 and 2, with the following exceptions. In the rating phase, the participants were presented with images of cosmetic products but not with the whole advertising images. Participants were asked to rate preference for the images of cosmetic products with a 5-point scale (1 = not preferable and 5 = preferable). The rating phase consisted of 16 trials. Half of the cosmetic products (8 trials) were included in the advertising images previously presented in the exposure phase (the exposed condition) whereas the remainder of the product images (8 trials) was not included in the exposed advertising images (the novel condition). The assignment of each set of product images to each exposure condition was counterbalanced across the participants. The presentation order of the product images was randomized for each participant.

### Results and Discussion

Two participants became aware of the purpose of the experiment and were excluded from the following analysis. As in Experiment 2, we calculated the *post hoc* power of the paired *t* test based on the effect size in Experiment 1, the sample size of this experiment, and significance level of α = 0.05. The obtained power was 0.80. This was considered sufficient to reliably identify the effect of mere exposure.

**Figure 2** shows the means of preference scores for the exposed and novel conditions. The paired *t* test did not show a significant difference between exposure and novel conditions, *t*(35) = 0.61, *p* = 0.55, *d*_z_ = 0.10, indicating no evidence of the mere exposure effect for the images of cosmetic products. This experiment had moderate power, and therefore, the absence of the mere exposure effect could not be attributed to the lack of statistical power. That is, this result suggests that when complex advertising images, such as those consisting of a product along with a female model, are repeatedly presented, the mere exposure effect does not always occur for each part of the advertising images.

To compare the amount of mere exposure effects between Experiment 2 and 3, a two-way mixed factorial ANOVA was conducted on preference scores with experiment (Experiment 2 versus Experiment 3) as a between-participant variable and exposure condition (exposed versus novel) as a within-participant variable. Results showed a significant interaction between experiment and exposure condition, *F*(1, 72) = 6.24, *p* = 0.01, *η_p_*^2^ = 0.08, indicating that a larger mere exposure effect was obtained in Experiment 2 than in Experiment 3.

## Experiment 4

In this experiment, we aimed to explore whether the absence of the mere exposure effect for the part of the advertisement with the product in Experiment 3 was partly caused because selective attention was captured by the female model. As discussed in the Introduction, many studies have demonstrated not only that participants’ attention tended to be captured involuntary by face stimuli, but also that participants could voluntary reallocate their attention to the other stimuli afterwards (Sui and Liu, 2009; Nakamura and Kawabata, 2014; Sato and Kawahara, 2015). Therefore, we expected that the mere exposure effect for the part of the advertisement with the product would be observed if participants were explicitly encouraged to direct their attention to that part in the exposure phase.

### Method

#### Participants

Thirty adults (16 males and 14 females) participated in this experiment (mean age = 19.97 years, *SD* = 1.25). None of these participants had participated in Experiments 1, 2, or 3. All participants had normal or corrected-to-normal vision.

#### Procedure

The procedure was identical to that of Experiments 3 except for the following additional instruction in the exposure phase. Participants were told that selective attention would be easily captured by the face stimuli, and that in this experiment they should exert themselves to direct their attention to the part of the advertisement with the product.

### Results and Discussion

As in Experiment 2 and 3, we calculated the *post hoc* power of the paired *t* test based on the effect size in Experiment 1, the sample size of this experiment, and significance level of α = 0.05. The obtained power was 0.72, which did not reach the criteria of 0.80 (Cohen, 1992). However, this might not have been problematic given that a large mere exposure effect was obtained from the manipulation of attention (Yagi et al., 2009), which was considered sufficient to find the reliable effect of mere exposure.

**Figure 2** shows the means of preference scores for the exposed and novel conditions. The paired *t* test showed that the mean preference score was higher in the exposed condition than in the novel condition, *t*(29) = 3.74, *p* < 0.01, *d*_z_ = 0.68, indicating that the mean preference score was higher in the exposed condition than in the novel condition. This result suggests that the mere exposure effect did occur for a product in an advertising image, if participants were to attend to the image in the exposure phase.

To compare the amount of mere exposure effects between Experiment 3 and 4, a two-way mixed factorial ANOVA was conducted on preference scores with experiment (Experiment 3 versus Experiment 4) as a between-participant variable and exposure condition (exposed versus novel) as a within-participant variable. Results showed a significant interaction between experiment and exposure condition, *F*(1, 64) = 7.83, *p* < 0.01, *η_p_*^2^ = 0.11, indicating that a larger mere exposure effect was obtained in Experiment 4 than in Experiment 3.

## General Discussion

The aim of the present study was to clarify in what part of an advertising image the mere exposure effect might occur. Experiment 1 was conducted to confirm the mere exposure effect for repeatedly presented advertising images. To this end, participants were repeatedly exposed to advertising images and were asked to rate preferences for the whole advertising image. The results showed the mere exposure effect (i.e., greater preference for the exposed advertising images than the novel ones). Experiments 2 and 3 were conducted to clarify whether the mere exposure effect occurs for the discrete parts of repeatedly presented advertising images. In Experiment 2, participants were asked to rate the preference for the attractive female models previously included in the advertising images. The results again showed the mere exposure effect (i.e., greater preference for the exposed models than for the novel ones). Interestingly, when participants were asked to rate the preference for the cosmetic products previously included in the advertising images (Experiment 3), the results showed no evidence for the mere exposure effect. The absence of the mere exposure effect for the product parts could be explained attentional capture by a female model. Consistent with this view, the mere exposure effect for the product parts was obtained when participants were encouraged to direct their attention to the product parts in the exposure phase (Experiment 4). In summary, we found that the mere exposure effect does not always occur for every part of the exposed advertising images and that attention would modulate the mere exposure effect for advertising images.

This finding contradicts the naïve view of the mere exposure effect that repeated exposure is sufficient to induce the positive change of attitude toward the repeatedly presented stimuli (Zajonc, 1968; Monahan et al., 2000). Instead, the results of Experiments 3 and 4 are consistent with the growing evidence that repeated exposure is not sufficient for the mere exposure effect (Lee, 2001; Craver-Lemley and Bornstein, 2006; Yagi et al., 2009; Stafford and Grimes, 2012; De Zilva et al., 2013; Inoue et al., 2018). Note that one major difference between Experiment 3 of this study and previous studies is the role of top-down instructions concerning how participants perceive stimuli in the exposure phase. Given that human faces, especially attractive faces spatially and temporally capture attention (Sui and Liu, 2009; Nakamura and Kawabata, 2014; Sato and Kawahara, 2015), it is reasonable to consider that the attractive female models in the advertising images captured participant’s attention in the exposure phase, simultaneously inducing relatively less attention to the cosmetic products. This inattention could lead to the absence of the mere exposure effect for the cosmetic products due to the importance of attention in the mere exposure effect (Yagi et al., 2009; Huang and Hsieh, 2013). According to this point of view, the present study could be considered as extending the implications of the study by Yagi et al. (2009) into the domain of consumer psychology. In this study, we do not propose that the mere exposure effect would never be observed for unattended objects (Bornstein, 1989). Rather, we are suggesting that attention facilitates the effect of repeated exposure (Yagi et al., 2009). Ideally, further studies are needed to explore the possibility that the mere exposure effect would occur for images of commercial products even without the presence of distracting stimuli such as female models.

The different mere exposure effects for the cosmetic products previously included in the advertising images has an important implication for the application of the mere exposure effect to advertisement. In general, sponsors of advertisement might expect that repeated presentation of advertising images for commercial products increase the positive attitude toward the commercial products, thereby raising the sales of the products. However, the results of Experiment 3 are inconsistent with this expectation. That is, the mere exposure effect did not occur for the cosmetic products even when advertising images including these products were repeatedly presented. On the other hand, the mere exposure effect was found for the same products when they were attended in the exposure phase. From the perspective of the mere exposure effect, these findings suggest that the use of advertising images consisting of a product with an attractive model might be ineffective for increasing the sales of the product, unless attention is directed to the product parts of repeated advertising images. This contention is consistent with the finding of a recent study on advertising by Bell and Buchner (2018). They reported a robust mere exposure effect for pop-up banners that were presented at the center of a display (even though they were evaluated as annoying), but not for static banners that were presented in the peripheral area of the display. It is well-known that pop-up stimuli capture attention (Jonides and Yantis, 1988). Therefore, it might be important to develop an effective design for advertising images in which an individual’s attention is involuntarily directed to the commercial product rather than to human models. One feasible method of achieving this would be to use the gaze cuing effect in which the gaze direction of a face stimulus directs observers’ attention to focus in the same direction (Driver et al., 1999). Based on this idea, certain studies have demonstrated that models in advertising images whose gaze is focused on commercial products enhanced the memory for those products (Hutton and Nolte, 2011; Sajjacholapunt and Ball, 2014). Further studies are needed to increase the effectiveness of the mere exposure effect on advertisement.

## Conclusion

When advertising images, consisting of a commercial product along with an attractive female model, were repeatedly presented, the mere exposure effect can occur for the female model without a specific instruction to direct participant’s attention to the female model whereas the mere exposure effect for the commercial product depends on the instruction for encouraging participants to direct their attention to the product. This suggests that repeated exposure to advertising images cannot always increase the sales of the products, and that attention would modulate the mere exposure effect for advertising images.

## Ethics Statement

The present study was approved by the Ethics Committee of Institute of Psychology at Rissho University. Informed consent was orally obtained from all participants.

## Author Contributions

All authors equally contributed to the design and implementation of this study and to the analysis of the results, as well as to writing the manuscript.

## Conflict of Interest Statement

The authors declare that the research was conducted in the absence of any commercial or financial relationships that could be construed as a potential conflict of interest.
